# Stromal Cells Induce Th17 during *Helicobacter pylori* Infection and in the Gastric Tumor Microenvironment

**DOI:** 10.1371/journal.pone.0053798

**Published:** 2013-01-24

**Authors:** Irina V. Pinchuk, Katherine T. Morris, Robert A. Nofchissey, Rachel B. Earley, Jeng-Yih Wu, Thomas Y. Ma, Ellen J. Beswick

**Affiliations:** 1 Department of Internal Medicine, Division of Gastroenterology and Hepatology, University of Texas Medical Branch, Galveston, Texas, United States of America; 2 Department of Surgery, University of New Mexico, Albuquerque, New Mexico, United States of America; 3 Department of Molecular Genetics and Microbiology, University of New Mexico, Albuquerque, New Mexico, United States of America; 4 Departement of Internal Medicine, Division of Gastroenterology, Kaohsiung Medical University, Sanmin District, Kaohsiung City, Taiwan; 5 Department of Internal Medicine, Division of Gastroenterology and Hepatology, University of New Mexico, Albuquerque, New Mexico, United States of America; University of Pittsburgh, United States of America

## Abstract

Gastric cancer is associated with chronic inflammation and *Helicobacter pylori* infection. Th17 cells are CD4^+^ T cells associated with infections and inflammation; but their role and mechanism of induction during carcinogenesis is not understood. Gastric myofibroblasts/fibroblasts (GMF) are abundant class II MHC expressing cells that act as novel antigen presenting cells. Here we have demonstrated the accumulation of Th17 in *H. pylori-*infected human tissues and in the gastric tumor microenvironment. GMF isolated from human gastric cancer and *H. pylori* infected tissues co-cultured with CD4^+^ T cells induced substantially higher levels of Th17 than GMF from normal tissues in an IL-6, TGF-β, and IL-21 dependent manner. Th17 required interaction with class II MHC on GMF for activation and proliferation. These studies suggest that Th17 are induced during both *H. pylori* infection and gastric cancer in the inflammatory milieu of gastric stroma and may be an important link between inflammation and carcinogenesis.

## Introduction

Gastric cancer is the 4^th^ most prevalent cancer with the 2^nd^ highest cancer-related mortality rates worldwide. The risk of developing gastric cancer is 1 in 115, with a survival rate of only 20–30% for 5 years [1]. Chronic inflammation associated with *Helicobacter pylori* infection is the number one risk factor for gastric cancer. *H. pylori* infects over 50% of the world's population with 1% of those infected going on to develop gastric cancer. An estimated 75% of all gastric cancer cases are associated with *H. pylori* infection [2]. The carcinogenic potential of *H. pylori* is driven by the interplay between bacterial virulence factors and the host's immune responses resulting in chronic inflammation, which in turn leads to tumorigenesis.

One of the major components of the immune system in inflammatory diseases that have been recently recognized are Th17 cells. Th17 cells are a CD4^+^ T cell phenotype that are characterized by expression of the RORγt transcription factor and IL-17 production. Th17 are associated with tissue damage in chronic inflammatory disorders such as Crohn's disease and rheumatoid arthritis [3], [4]. IL-17A induces the production of pro-inflammatory cytokines from other cell types, particularly IL-8 production from gastric epithelial cells (GECs), which is another important cytokine in *H. pylori* host responses [5]. An increase in IL-17 during *H. pylori* infection is suggested to be responsible for mucosal damage during gastritis and cytokines associated with Th17 have damaging tumor promoting effects in multiple cancers [5]–[7]. However, how this T helper cell phenotype develops in the stomach and the role it plays in disease outcome remains unclear.

Th17 are known to develop in the presence of IL-6 and TGF-β [8]. Additionally, development and maintenance of this phenotype may further require IL-21, IL-23, or IL-1β, suggesting that there are several mechanisms that lead to the development of Th17 [9], [10]. We have previously shown that GECs express cytokines and receptors that influence the T cell response during *H. pylori* infection [11], [12]. In addition to GEC, we have also shown that CD90^+^ stromal cells (myofibroblasts and fibroblasts, MF) act as antigen presenting cells in the colon [13], [14]. However, the ability of gastric MF (GMF) to impact CD4^+^ T cell differentiation and proliferation in gastric tissue has not been examined. A closer look at the impact of MF is crucial since MF in other systems have been shown to impact inflammation, carcinogenesis, and metastasis. The aim of this study was to investigate the role of GMF in Th17 development in human tissues obtained from *H. pylori* infected and gastric cancer patients. MFs are important players in the tumor microenvironment and are thought to play a crucial role in inflammation and carcinogenesis by releasing growth factors that act on tumors [15], [16]. Data from the past decade indicate that MF are key regulators of chronic inflammation in peripheral organs, and upon inflammation MF display a fundamentally altered phenotype, differentially impacting epithelial cells in a paracrine manner [17], [18].

This work utilizes normal, *H. pylori* infected, and gastric cancer human tissues to examine the role of GMF in the increased Th17 response seen in *H. pylori* associated gastric inflammation and cancer. Our data confirm the increase in Th17 in *H. pylori* infection and in human gastric tumors, and further show *H. pylori* primed GMF promote differentiation of Th17. This process was IL-6, TGF-β, and IL-21 dependent, which *H. pylori-*exposed and gastric tumor derived MF produced at increased levels, thus maintaining a stronger ability to induce Th17 cells. Our findings suggest that the enhanced Th17 promoting capacity of the GMF derived from gastric tumors may be among the key factors contributing to gastric tumor promoting inflammatory milieu.

## Methods

### Tissue, Cells and Bacteria Culture

All human samples were collected under IRB approved human protocols at the University of New Mexico Health Sciences Center, Legacy Research Tumor Bank, and Kaohsiung Medical University. Written consent was obtained with IRB approved consent forms from each institution. Primary cultures of MF from 3 sets of matched normal and cancer GMF were attained by EDTA treatments and dissociation using the GentleMACS system (Miltenyi Biotech, Bergisch Gladbach, Germany) and cultured as previously described [13], [14]. The purity of MF was analyzed by staining for CD90 and α-smooth actin (α-SMA) for flow cytometry. Gastric biopsies were obtained from healthy individuals and those with gastritis under an approved human protocol at UNM Health Sciences Center. *H. pylori* status was obtained from patient medical records. *H. pylori* strain 51B was grown on blood agar plates (Becton Dickinson, San Jose, CA) at 37°C under microaerophilic conditions. GMF were incubated with 50∶1 bacteria:cell ratio of *H. pylori* for 24 hours.

### Confocal Microscopy

GMF were grown on Millicell EZ slides, fixed with 1% paraformaldehyde, blocked with murine serum and stained with anti-α-smooth actin (clone 1A4 Sigma, St Louis, MO) AlexaFlour 488 conjugated using Zenon labeling kit (Life Technologies, Carlsbad, CA), and phycoerythin (PE) conjugated anti-HLA-DR (clone L243, BD Biosciences, CA) antibodies for 1 hour at room temperature. Staining was followed by six washes and samples were then mounted in SlowFade® Gold anti-fade reagent with DAPI (Life Technologies). Confocal microscopy was performed with a Zeiss LSM510 META laser microscope (Carl Zeiss, Thornwood, NY).

### Naïve CD4^+^ T cell Isolation and Incubation with GMF

Heparinized venous blood was obtained from healthy adult volunteers who signed written consents approved by the UNM IRB. Peripheral blood mononuclear cells (PBMC) were isolated by density gradient centrifugation over Ficoll-Paque Plus. Naïve CD4^+^ T cells were isolated from the PBMC using a negative selection kit (Stem Cell Technologies, Vancouver, WA) and purity examined by flow cytometry. These cells were mixed with anti-CD3/anti-CD28 coupled T cell activation beads (Life Technologies). Supernatants from *H. pylori-*treated cultures were filtered through syringes with 0.2 µm filters to remove bacteria and cells were washed 3 times with media to remove attached bacteria before adding T cells and conditioned media from infected cells. Neutralizing antibodies for TGF-β1 (Thermo Scientific, Waltham, MA), IL-6, IL-21, (1 µg/ml each) or isotype control (eBioscience) were added to GMF for 30 minutes before addition of T cells. Co-cultures were incubated for 48 hours at 37°C.

### Flow Cytometry

GMF were stained with PE conjugated anti-CD90 clone Thy-1 and anti-HLA-DR clone L243 (Biolegend) according to manufacturer's instructions, washed twice in PBS, and fixed in 2% paraformaldehyde. T cells were stained with anti-RORγt antibody, clone AFKJS-9 (eBioscience) PE or APC conjugated, using the FoxP3 staining buffer kit according to manufacturer's instructions. For analysis of proliferation, T cells were labeled with carboxyfluorescein succinimidyl ester (CFSE) from Life Technologies according to manufacturer's instructions, activated with anti-CD3/anti-CD28 coupled beads, and collected at Day 5 for analysis on a Guava easyCyte 8HT flow cytometer (Millipore, Bellerica, MA), and analyzed using FCS Express software (DeNovo Siftware, Los Angeles, CA).

### Luminex Arrays

Supernatants were analyzed for IL-17A, TGF-β, IL-6, and IL-21 using Milliplex kits (Millipore) according to manufacturer's instructions and analyzed on a Luminex 200.

### Real Time PCR

Human tissues from the UNM Human Tissue Repository and Legacy Research Tumor Bank were homogenized in trizol (Life Technologies) using the GentleMACS system. RNA was extracted according to manufacturer's instructions, readings taken with a Nanodrop (Thermo Fisher Scientific), and cDNA made using cDNA supermix kit (Quanta Biosciences, Gaithersburg, MD). cDNA was also obtained from Kaohsiung Medical University from tissues collected under institutional approved human protocols with approved written consent. Gene expression assays from Applied Biosystems (Foster City, CA) were used with PerfeCTa qPCR Supermix (Quanta Biosciences) to perform qPCR with an Applied Biosystems StepOne Plus machine with their two-step protocol. Quantification of gene expression was performed using the comparative CT method and reported as the fold difference relative to 18S mRNA.

### Statistical Analysis

Results were expressed as the mean ± SE of data obtained from at least three independent experiments done with triplicate sets in each experiment. Differences between means were evaluated by ANOVA using Student's *t*-test for multiple comparisons. Values of *P*<0.05 were considered statistically significant. Pearson's correlations were performed in GraphPad Prism 5.

## Results

### Th17 are increased in H. pylori infected and gastric cancer patient tissues

**Figure 1:**
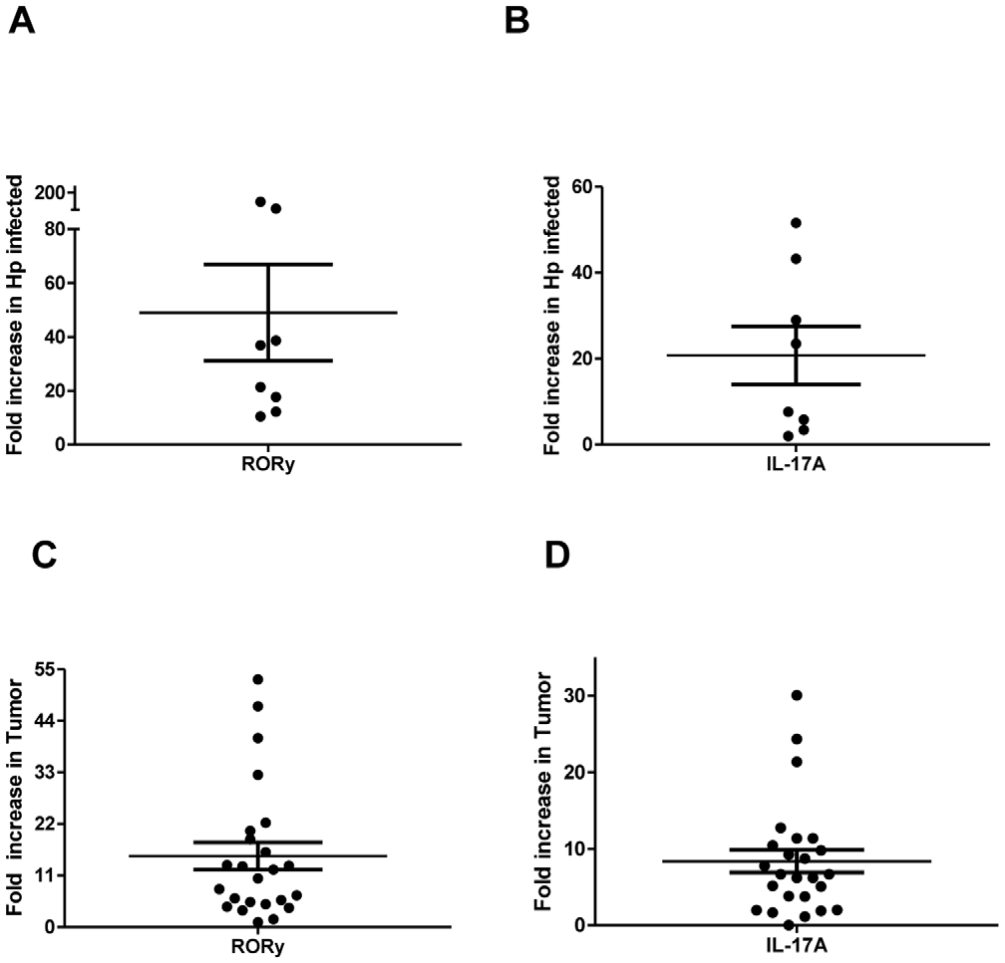
Th17 are increased in human tumor and *H. pylori*-infected tissues samples. Quantitative real time RT-PCR data indicates *H. pylori* infected tissues compared to unmatched normal tissues have increases in **A**) RORγ and **B**) IL-17A mRNA levels. Human gastric tumor samples compared to matched normal samples also have increased **C**) RORγ and **D**) IL-17A. The mRNA levels were normalized to 18S. Data represent mean of mRNA fold increase ± standard errors from triplicates compared to the levels in normal samples. * *p*<0.05.

Although *H. pylori* infection and gastric cancer are associated with chronic inflammation, little is known about the role Th17 play in the mucosa during these diseases. Thus, we examined the presence of the Th17 using RORγ and IL-17A markers in mucosa derived from normal samples, *H. pylori* infected samples with gastritis, and matched normal and gastric cancer tissues from the same patient. *H. pylori* infected mucosa samples were compared to the samples derived from *H. pylori* negative non-inflamed healthy controls, while we were able to obtain matched normal tissues from cancer patients. Five of the tumor samples were from patients confirmed to have previous *H. pylori* infections; however, *H. pylori* status was unknown for 14 samples. *H. pylori* infected samples showed a 10–200 fold increase in RORγ and 2–52 fold increase in IL-17A mRNA in all samples examined as shown by quantitative real time RT-PCR (**Figure 1A and 1B**). Moreover, in gastric cancer tissues, RORγ was upregulated 2–54 fold compared to matched normal samples in 23 of 24 samples examined, and IL-17A mRNA was increased 2–30 fold (**Figure 1C and 1D**). These results suggest that Th17 accumulate in gastric tumors and during *H. pylori* infection and produce IL-17A.

### GMF characterization and class II expression

**Figure 2:**
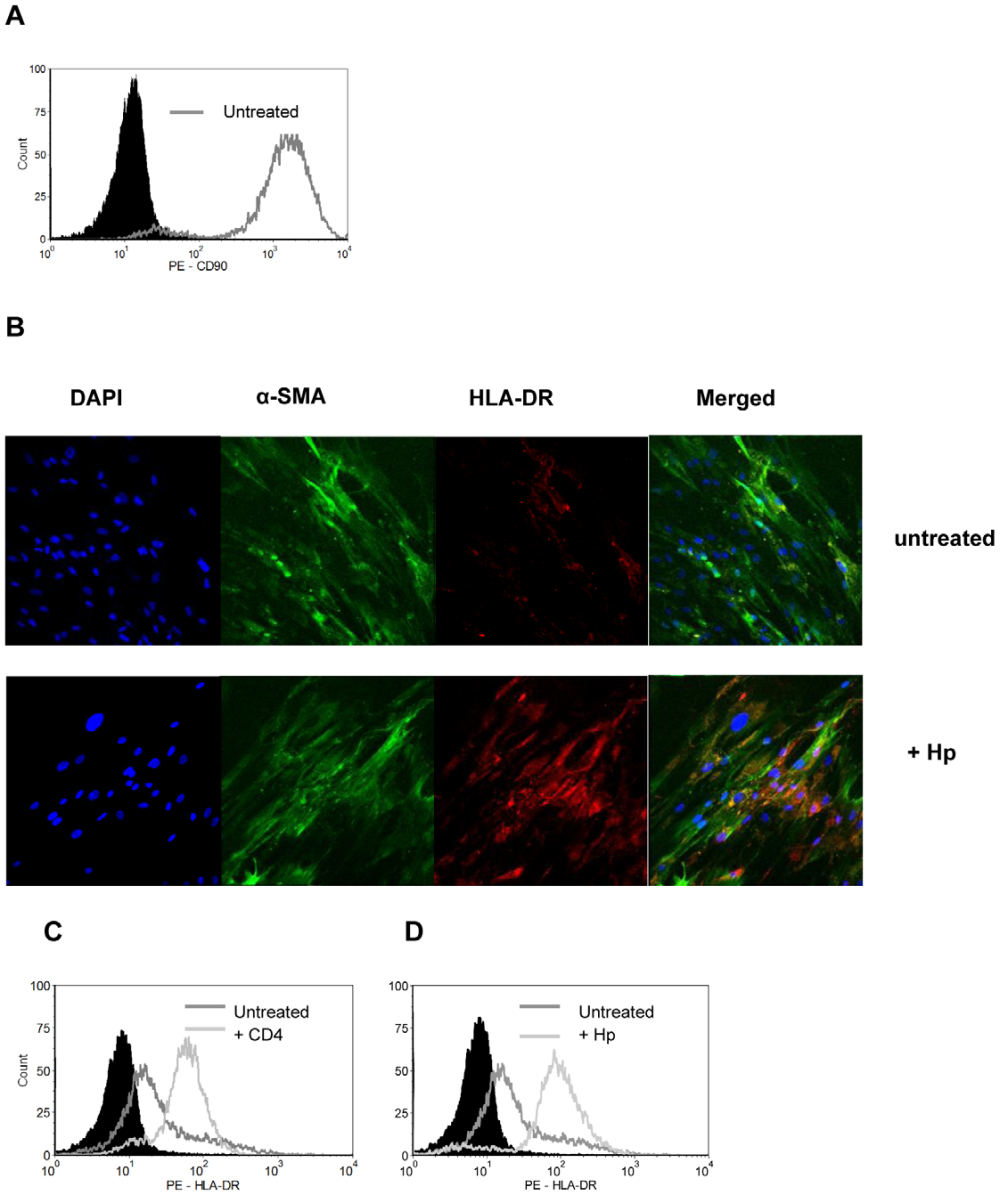
Gastric myofibroblast express CD90, α-smooth muscle actin, and class II MHC. Gastric myofibroblast express **A**) CD90 by flow cytometry compared to the solid peak isotype control. These cells also express **B**) α-smooth actin and class II MHC, where class II MHC is upregulated by exposure to *H. pylori* as shown by confocal microscopy. Class II MHC is also upregulated by **C**) incubation with CD4^+^ T cells and **D**) *H. pylori* exposure by flow cytometry. Figures are representative of 3 experiments in duplicate.

In order to investigate the potential of GMF to influence CD4^+^ T cell responses, GMFs were isolated from human gastric tissue biopsies or surgical resections and examined by flow cytometry and confocal microscopy for purity. In **Figure 2A**, >98% of isolated cells were found to express the fibroblast marker, CD90. Myofibroblasts, or activated fibroblasts, further express α-SMA, and 90–95% of isolated cells express this marker in our cultures (**Figure 2B**). Many tumors, including gastric tumors, are known to have increased amounts of myofibroblasts [19], [20]. However, we were able to culture similar amounts of α-SMA cells from normal and tumor tissues. Since it has been shown that *H. pylori* disrupts barrier function, infection may affect GMF as well as epithelium [21]. Hence, we found the class II MHC molecule, HLA-DR to be upregulated by *H. pylori* on these cells (**Figure 2B**). Moreover, class II MHC expression was also found to be increased on GMF cell surfaces after incubation with naïve CD4^+^ T cells (**Figure 2C**) and by *H. pylori* exposure by flow cytometry (**Figure 2C and 2D**). The expression and upregulation of class II MHC expression on GMF suggests that these cells may interact with T cells and influence their responses.

#### H. pylori infected and cancer derived GMF promote RORγt and IL-17A expression in CD4+ T cells

**Figure 3:**
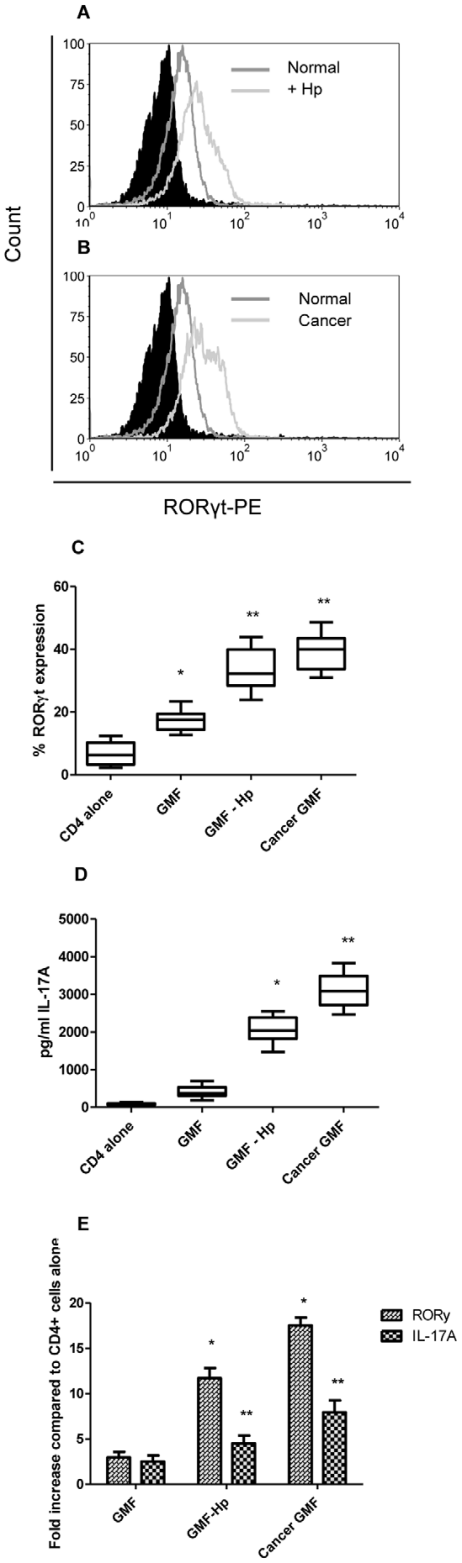
Th17 development increases in culture with *H. pylori-*exposed and cancer gastric myofibrobroblasts. **A**) N-GMF and Hp-GMF primed CD4^+^ T cells were analyzed for RORγt expression by flow cytometry. **B**) N-GMF and C-GMF primed CD4^+^ T cells were analyzed for RORγt expression by flow cytometry. **C**) Compiled data for all experiments of N-GMF, Hp-GMF, and C-GMF primed CD4^+^ T cells analyzed for RORγt expression by flow cytometry. **D**) IL-17A levels were measured in co-cultures of CD4^+^ T cells with GMF by Luminex bead array. The RNA levels from GMF primed CD4^+^ T cells were also analyzed by quantitative real time PCR for **E**) RORγ and IL-17A. The mRNA levels were normalized to 18S. Data represent mean of mRNA fold increase ± standard errors from three duplicate experiments from 3 sets of GMF (n = 18). * *p*<0.05, between CD4^+^ and CD4^+^ incubated with GMF, ** *p*<0.05 between untreated and *H. pylori* treated or cancer.

We and others previously demonstrated that intestinal derived CD90^+^ stromal cells produce several cytokines that are involved in T cell differentiation upon stimulation with pro-inflammatory stimuli [22], [23]. Thus, we examined whether GMFs isolated from normal (N-GMF), *H. pylori*-exposed (Hp-GMFs), and tumor tissues (C-GMFs) may promote Th17 cell phenotype. Naïve CD4^+^ T cells were negatively selected with a >98% purity (data not shown), activated with anti-CD3/anti-CD28 coupled beads, incubated with GMF for 48 hours, stained with RORγt, and analyzed by flow cytometry. As seen in **Figure 3A and 3B**, RORγt expression by CD4^+^ T cells was increased in cells incubated with Hp-GMF and C-GMF compared to N-GMF. Compiled data from multiple experiments indicates that RORγt was expressed at an average of 7% in activated CD4^+^ T cells alone, but when incubated with N-GMF increased to 17% (**Figure 3C**). N-GMF were exposed to *H. pylori* for 24 hours prior to addition of CD4^+^ T cells to cultures, *H. pylori* was removed by washing cells multiple times, and *H. pylori* was filtered from the media to make *H. pylori* conditioned media. When CD4^+^ T cells were incubated with these *H. pylori*-exposed GMF, RORγt expression was increased to 33% and with C-GMFs to 39%. IL-17A production was also examined in co-cultures by Luminex bead array and found to be increased in supernatants by 5 and 8 fold respectively when T cells were primed with *H. pylori*-exposed N-GMF and C-GMFs, when compared to N-GMFs (**Figure 3D**). RORγ and IL-17A RNA levels were also increased in T cells, up to 17 fold and 7 fold, respectively, when incubated with *H. pylori*-exposed N-GMF and C-GMF compared to N-GMF (**Figure 3E**). These results suggest that not only are Th17 accumulating in gastric tissues during infection and cancer, but also may differentiate in the gastric mucosa by interacting with GMF.

#### IL-6 and TGF-β are upregulated in H. pylori infected human tissues and in cancer GMFs and promote Th17 development

**Figure 4:**
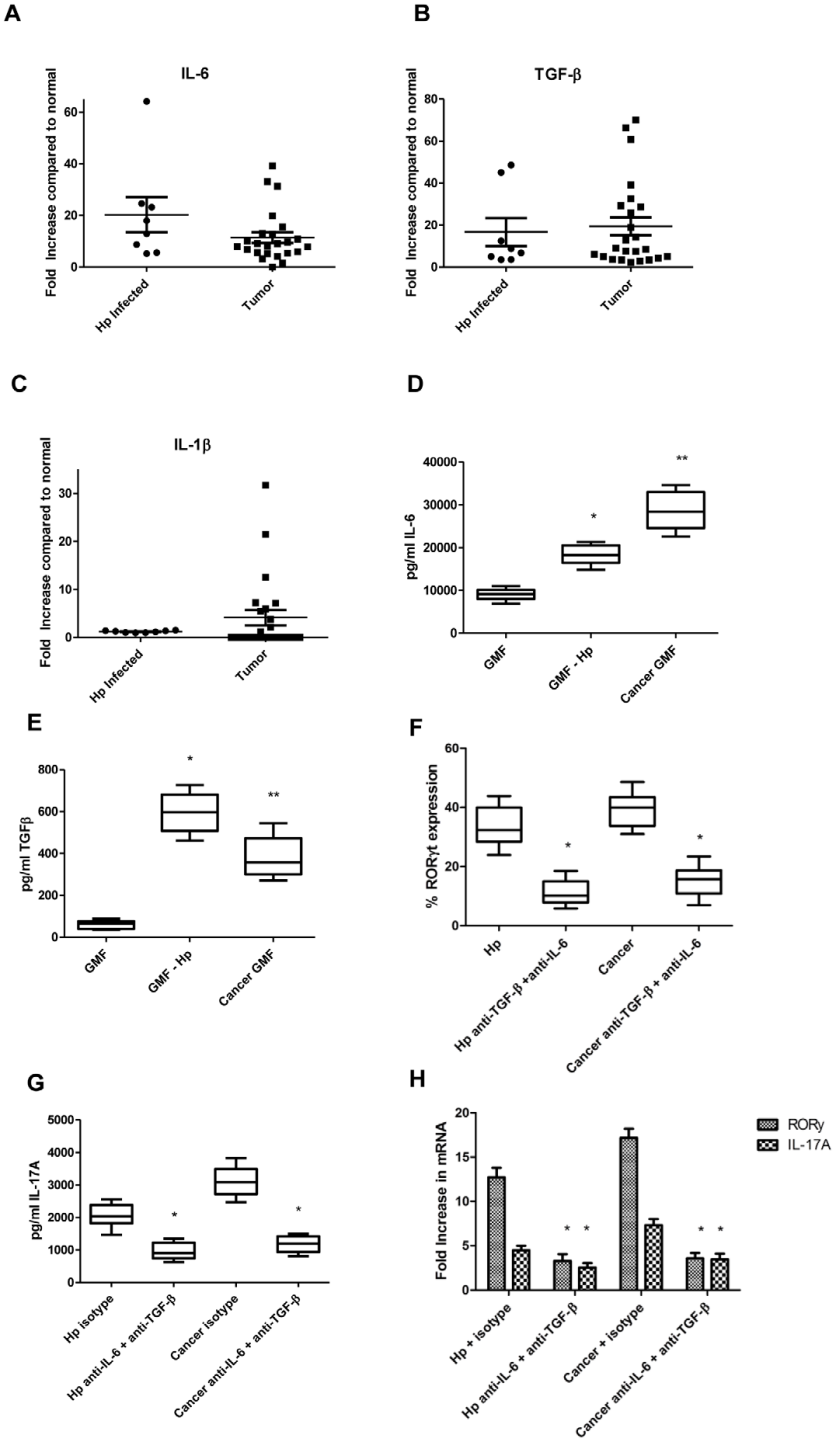
IL-6 and TGF-β produced in tumors and by GMF contribute to Th17 development. The level of mRNA measured by quantitative real time RT-PCR in *H. pylori* infected and cancer tissues compared to normal tissues for **A**) IL-6, **B**) TGF-β, and **C**) IL-1β. Data represent mean of mRNA fold increase ± standard errors from triplicates compared to the levels in matched normal samples. Supernatants from GMF cultures were analyzed for **D**) IL-6 and **E**) TGF-β by Luminex bead array. **F**) RORγt expression by GMF primed CD4^+^ T cells was analyzed by flow cytometry. **G**) IL-17A production was measured in supernatants by Lunimex bead array. **H**) RORγ and IL-17A mRNA levels in cancer and *H. pylori-*exposed GMF primed CD4^+^ T cells was examined by quantitative real time RT-PCR, normalized to 18S, and compared to normal GMF. Data represent mean of mRNA fold increase ± standard errors from three duplicate experiments in triplicate from 3 sets of GMF (n = 18). * *p*<0.05 between normal and *H. pylori* treated or cancer, ** *p*<0.05 *H. pylori* treated or cancer and TGF-β and IL-6 neutralization.

Since IL-6, TGF-β, and IL-1β are suggested to be required for Th17 development, we examined the mRNA of these cytokines in human tissues. In *H. pylori* infected tissues and gastric tumor tissues, gene expression was examined by quantitative real time RT-PCR. IL-6 was increased between 5 and 64 fold in all *H. pylori* infected samples and 22 of 24 tumor samples up to 39 fold (**Figure 4A**). TGF-β was increased between 3 and 49 fold in all *H. pylori* infected samples compared to normal samples and up to 66 fold in all tumor samples (**Figure 4B**). IL-1β was not increased in *H. pylori* infected samples, but was increased in 10 of 24 tumor samples up to 31 fold when compared to normal tissues (**Figure 4C**). As seen in **Table 1**, Pearson's correlations were used to show a strong relationship between IL-6 and TGF-β and the RORγ and IL-17A values shown in **Figure 1**, but only a moderate relationship was seen with IL-1β in cancer tissues and this was only found in less than half of the samples. In GMFs isolated from human samples, there was an increase in IL-6 production from 9038 pg/ml from N-GMF to 18,230 pg/ml with Hp-GMF and 28,470 pg/ml with C-GMF (**Figure 4D**). TGF-β production was also increased from 61 pg/ml with N-GMF to 599 pg/ml with Hp-GMF and 398 pg/ml with C-GMF (**Figure 4E**). Since these cytokines are known to both be needed for Th17, both were neutralized together with monoclonal antibodies. Single blocking experiments were conducted (not shown), but neutralizing both cytokines was needed to get maximal decreases in RORγt expression. When both were blocked RORγ expression in T cells was decreased by approximately 60% in both Hp-GMF and C-GMF (**Figure 4F**). IL-17A production was increased as shown by Luminex bead array in parallel to RORγt expression (**Figure 4G**). Real time PCR analysis also revealed substantial decreases in RORγ and IL-17A mRNA levels when IL-6 and TGF-β were neutralized (**Figure 4H**). These combined data suggest that production of the cytokines essential for Th17 development is upregulated in *H. pylori* infection exposure and cancer in GMF, suggesting a novel mechanism for local differentiation of Th17 in disease affected gastric mucosa.

**Table 1:** Pearson's Correlations.

	Hp infected	Hp infected	Tumor	Tumor
	RORγ	IL-17A	RORγ	IL-17A
**IL-6**	0.8232	0.8863	0.9118	0.9290
**TGF-β**	0.9551	0.7979	0.8278	0.8447
**IL-1β**	N/A	N/A	0.5649	0.3046
**IL-21**	0.8123	0.7559	0.7893	0.6818
**IL-23**	0.3142	0.2327	0.5490	0.2137

#### IL-21 plays a role in Th17 development and is present in human H. pylori infected and gastric cancer tissues

**Figure 5:**
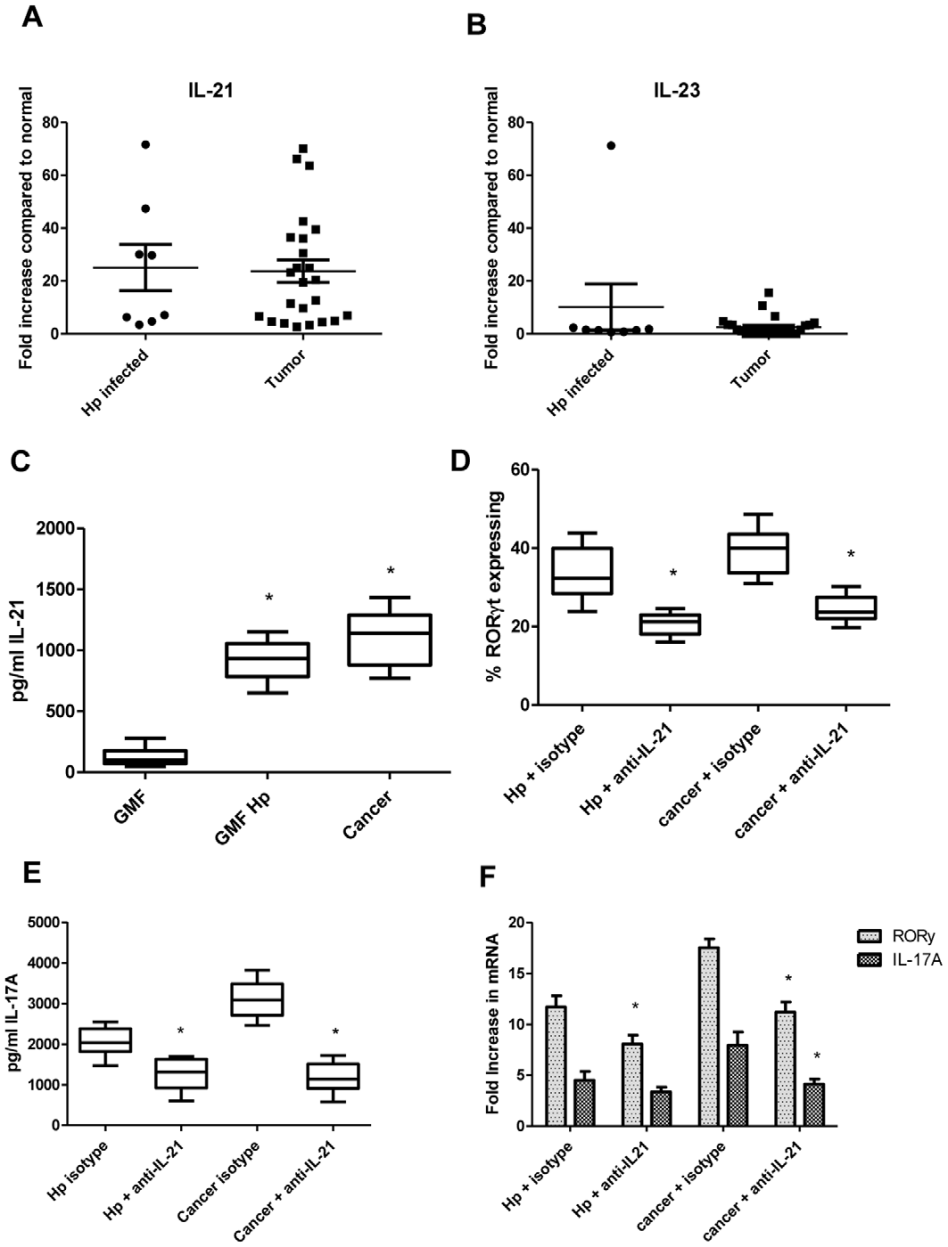
IL-21 produced in tumors and by T cells in culture with GMF contributes to Th17 development. **A**) IL-21 and **B**) IL-23 mRNA levels measured by quantitative real time RT-PCR in *H. pylori*-infected tissues and in human tumors compared to normal tissues when mRNA levels were normalized to 18S. Data represent mean of mRNA fold increase ± standard errors from triplicates compared to the levels in matched normal samples. Supernatants from CD4^+^ T cell/GMF co-cultures were analyzed for **C**) IL-21 by Luminex bead array. **D**) RORγt expression by GMF primed CD4^+^ T cells was analyzed by flow cytometry. **E**) IL-17A levels were measured in supernatants by Luminex bead array. **F**) RORγ and IL-17A mRNA levels in cancer and *H. pylori* exposed GMF primed CD4^+^ T cells was examined by quantitative real time PCR, normalized to 18S, and compared to normal GMF. Data represent mean of mRNA fold increase ± standard errors from three experiments in duplicate from 3 sets of GMF (n = 18). * *p*<0.05 between normal and *H. pylori* treated or cancer or *H. pylori* treated or cancer and IL-21 neutralization.

Th17 development and maintenance of this phenotype has been suggested to further require IL-21 and/or IL-23. Thus, these cytokines were examined in human gastric tissues by quantitative real time RT-PCR. In *H. pylori* infected samples, IL-21 was increased in all samples from 3 to 72 fold, and IL-23 was found to be increased in only 2 samples, one by 2 fold and one by 72 fold (**Figure 5A and 5B**). All 24 tumor samples had an increase in IL-21 mRNA levels, up to 70 fold, while only 12 of 24 samples had an increase in IL-23 mRNA levels, up to 15 fold, compared to matched normal tissue. Pearson's correlation (**Table 1**) demonstrates a strong positive correlation between RORγ and IL-17A with IL-21in both Hp-infected tissues and cancer tissues. There was a low to moderate correlation with IL-23 as well, but it was expressed in only 50% of the samples. IL-21 was also found in co-culture supernatants at similar levels for *H. pylori*-exposed and cancer GMF as measured by Luminex bead array (**Figure 5C**); however, IL-23 was not found in supernatants (not shown). Neutralizing IL-21 also decreased T cell expression of RORγt by flow cytometry and IL-17A in a Luminex bead array (**Figure 5D–E**), but with less magnitude than neutralizing IL-6 and TGF-β as shown in **Figure 4F**. Parallel real time RT-PCR data shows similar changes in RORγ and IL-17A mRNA levels (**Figure 5F**). These data supports the role for IL-21 in Th17 development in *H. pylori* infection and gastric cancer, although IL-23 was not required here.

#### Th17 that develop from GMF proliferate in a class II MHC dependent manner

**Figure 6:**
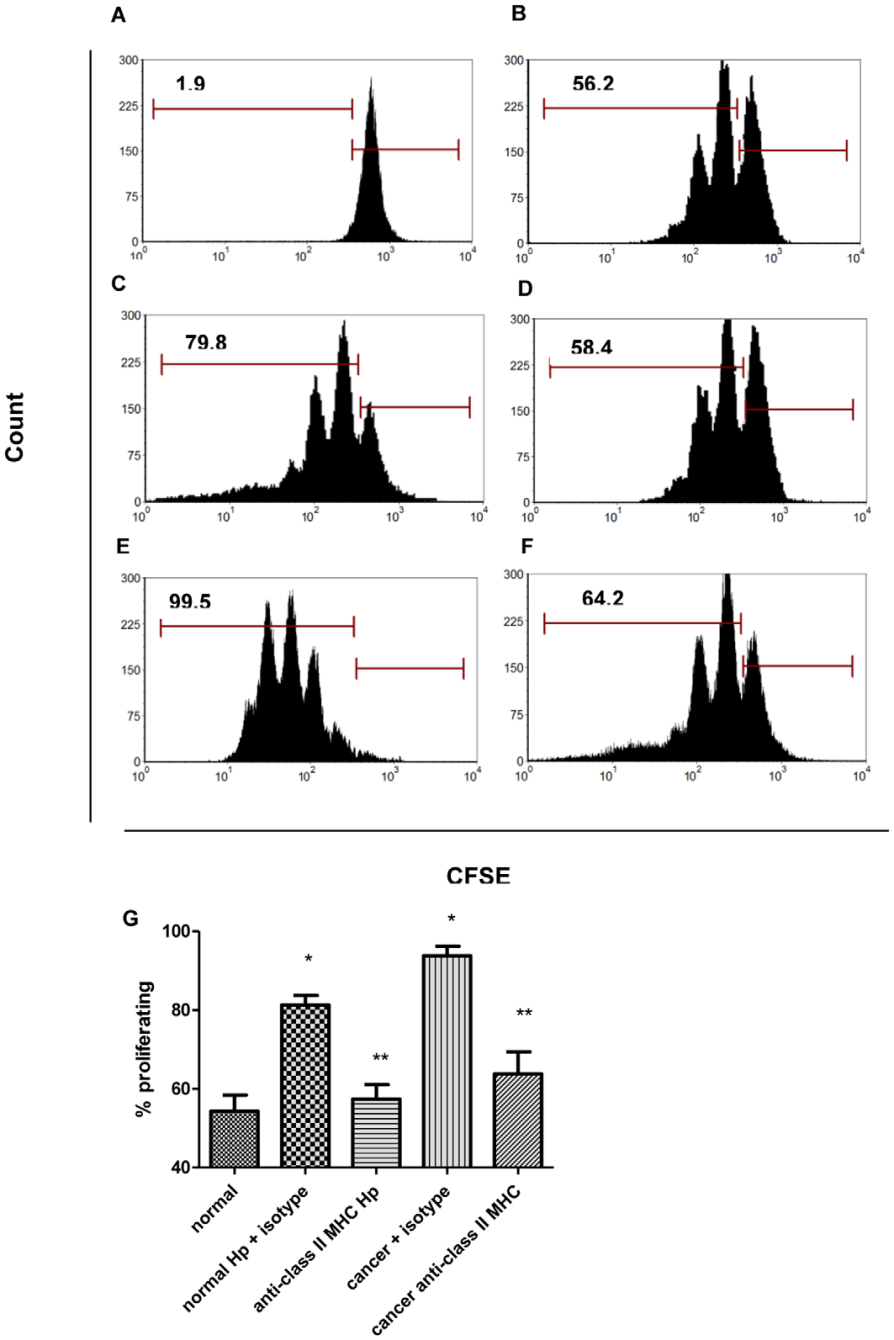
RORγt expressing cells proliferate in culture with GMF in a class II MHC dependent manner. CD4^+^ T cells labeled with CFSE and gated on RORγt^+^ cells for representative histograms for **A**) non-proliferating control, **B**) CD4^+^ T cells in culture with normal GMF **C**) CD4^+^ T cells in culture with *H. pylori-*exposed GMF, **D**) CD4^+^ T cells in culture with *H. pylori-*exposed GMF pre-incubated with class II MHC blocking antibodies **E**) CD4^+^ T cells in culture with cancer GMF **F**) CD4^+^ T cells in culture with cancer GMF pre-incubated with class II MHC blocking antibodies. **G**) Compiled data from 3 experiments in triplicate show the percent proliferating cells from CD4^+^ T cells in culture with normal GMF, *H. pylori-*exposed GMF, and cancer GMF with class II MHC blocking. (n = 9). * *p*<0.05 *H. pylori* treated or cancer and class II MHC blockade.

Next we sought to examine if the Th17 developed in culture with GMF were actively proliferating. CFSE labeled, anti-CD3/anti-CD28 activated CD4^+^ T cells were cultured with GMF for 5 days. Cells were stained for RORγt and gated on RORγt expressing cells. *H. pylori-*exposed and cancer derived GMF priming of T cells resulted in an increased rate of Th17 proliferation compared to those incubated with N-CMFs. However, when class II MHC was blocked, the proliferation of CD4^+^ T cells in culture with C-GMF was decreased to similar levels as N-GMF (**Figure 6A–F**). **Figure 6G** shows compiled data from CD4^+^ T cells co-cultured with N-GMF, Hp-GMF, and C-GMF. Blocking class II MHC on both Hp-GMF and C-GMF led to a marked decrease (up to 40%) in proliferation. This decrease indicates that not only do Th17 require cytokines for development, but also require contact with class II MHC on GMF to sustain proliferation. When class II MHC was blocked to examine development of Th17, there was no measurable change in RORγt or IL-17A expression (not shown) demonstrating that IL-6, TGF-β, and IL-21 are a required mechanism for Th17 development, while class II MHC is required for the maximum proliferative potential.

## Discussion

A link between *H. pylori* infection, chronic inflammation, and carcinogenesis has long been suggested. However, host responses to *H. pylori* infection are complex and the elements of the local immune system promoting chronic inflammation and carcinogenesis are unclear. Th17 cells may be an important immune response component triggering development of gastric carcinogenesis during *H. pylori* associated chronic inflammation. The relevance of the IL-17 to *H. pylori* associated inflammation has been proven using animal models where *H. pylori* infection led to upregulation of IL-17 in gastric tissue and its neutralization or knockdown resulted in decrease of mucosal inflammation and bacterial load [24]. The recruitment of Th17 early in infection was also associated with inflammation and an increased disease state [25]. Th17 responses have been shown to promote tumor growth in various cancers, such as breast cancer, cervical cancer, and melanoma, although other studies suggest a protective role for these cells [26]–[29]. The limited studies thus far in gastric cancer suggest that increased Th17 accumulation in the gastric tumor microenvironment is linked to disease progression and poor outcome. A recent study revealed that increased levels of Th17 associated factors in gastric cancer patients in peripheral blood and tumor draining lymph nodes correlated with advanced disease [30]. Other studies also supported this notion by demonstrating that Th17 in circulation were associated with tumor progression and are a potential prognostic factor for poor outcome [31]–[34]. Although the role of Th17 in cancer development remains controversial, no study to date has suggested that these cells are protective in gastric cancer. These recent reports led us to examine the presence of Th17 cells in normal, infected and cancer samples. We found that RORγ and IL-17A, the main Th17 factors, are highly upregulated in gastric mucosa derived from both *H. pylori* infection and gastric cancer suggesting a link between *H. pylori* infection, the inflammatory CD4^+^ T cell phenotype, Th17, and gastric cancer.

Despite the advance in the understanding of the role of Th17 cells in *H. pylori* associated chronic inflammation and carcinogenesis, the mechanisms and cells responsible for the increase in the Th17 responses remain unclear. It has been recently demonstrated that professional antigen presenting cells such as monocyte-derived dendritic cells and macrophages induce IL-17 production by CD4^+^ T cells upon *H. pylori* infection [35], [36]. In contrast, the contribution of the mucosal non-professional APCs, such as GEC and GMFs is unknown, although they are very prevalent class II MHC expressing cells in the gastric mucosa. Fibroblasts represent a crucial component of the cancer microenvironment and inhibition of the activation of these cells leads to a decrease in tumor size in breast and colon cancer animal models. Since both myofibroblasts and carcinoma cells produce cytokines and growth factors that may act in a paracrine manner on adjacent cells, tumor cells may also activate stromal fibroblasts as shown by one group in a mouse model of gastric cancer and another group with human colorectal stromal cells and tumor cells [37], [38]. Moreover, there is an emerging concept that CD90^+^ stromal cells are among the key regulators of acute and chronic inflammation [17], [18]. These cells are found in close association with T cells in peripheral mucosa and lymphoid organs [39], [40] and we have demonstrated that colonic fibroblasts express MHC class II molecules and modulate CD4^+^ T cell activity [13], [14], while others have shown a role for dermal fibroblasts in Th17 development [23]. Thus, examining GMF is necessary to understand the T cell responses in the gastric mucosa. Moreover, contact between *H. pylori* and stromal cells in mucosa is plausible because the mucosal barrier is disrupted during infection, gastritis, and ulceration leading to the transit of bacterial material and entire bacteria from lumen to the sub-epithelial mucosal layer [41], [42]. Thus, our study supports the hypothesis that GMFs contribute to the Th17 increase observed during *H. pylori*-associated chronic inflammation and gastric cancer.

We demonstrated that co-culture of N-GMFs with *H. pylori* or pre-activated CD4^+^ T cells resulted in a strong upregulation of class II MHC molecules by GMFs. These data combined with our previous data reporting the APC capacity of colonic CD90^+^ stromal cells [13], [14] suggests that GMFs may serve as local APCs in gastric mucosa. Moreover, Hp-GMF and C-GMF induced significantly higher Th17 responses than N-GMF. Further, we demonstrated that the GMF-mediated induction of Th17 occurred via IL-6 and TGF-β, which are not only increased by C-GMF, but are also important cytokines in both Th17 development and in tumor development. Th17 generation in the co-culture with GMFs required increased IL-21 production by T cells. Interestingly, we also found sizeable increases in IL-21 in human gastric cancer mucosa compared to normal, which is in agreement with work by Iida et al. [31]. However, only 50% of the tumor samples had increased IL-1β and IL-23, which are also important in Th17 development and maintenance. We did not find these cytokines in culture, suggesting that IL-6, TGF-β, and IL-21 are sufficient for Th17 development in our system. However, IL-1β and IL-23 are likely produced by other cells and may further support development of Th17 as seen in other studies of both *H. pylori* infection and cancer [43], [44]. Moreover, we did not exclude that GMFs can indirectly contribute to the IL-1β and IL-23 dependent Th17 upregulation in the gastric mucosa since human skin derived fibroblasts were demonstrated to support the expansion of Th17 cells via upregulation of the IL-23 production by dendritic cells via a process involving IL-1β [23]. Since DCs are important in Th17 development, the role of these cells in *H. pylori* infection and gastric cancer should be further examined. One group has suggested that DCs lead to regulatory T cell development and suppression of Th17 during *H. pylori* infection [45], while others have suggested a Th17 dominant response in the mouse model of infection so the role of these cells is not clear.

Importantly, we demonstrated that Hp-GMFs and C-GMFs also strongly support proliferation of Th17 cells, when compared to N-GMFs or T cell alone. The increase in the Th17 proliferation promoting capacity can be correlated with class II MHC expression on GMFs and was decreased upon blocking class II MHC. The importance of class II MHC molecules in the expansion of the IL-17 producing T cells was also noted during the pathogenesis of multiple sclerosis and type I diabetes [46], [47]. Thus, taken together with earlier findings, our data suggest that Th17 require contact with APCs for full proliferation potential. Although the importance of stromal cells in cancer and infection associated inflammation is well established, the exact role of these cells is far from understood. For the first time, our data showed CD90^+^ stromal cells to be among the key cells responsible for the generation of local Th17 responses in both *H. pylori* infection and gastric cancer. Importantly, C-GMFs represent a stable Th17 promoting phenotype similar to that observed upon *H. pylori* infection of N-GMFs. The commonality of Th17 accumulation in both *H. pylori* infection and gastric cancer should be examined for a link between chronic inflammation and carcinogenesis. Our and other laboratories report that under chronic inflammatory conditions stromal cells produce high levels of disease-promoting molecules without further stimulation by immune cells. In the colon, we previously reported that normal MF give rise to regulatory T cells, but upon inflammation their capacity to induce inhibitory T cells is diminished [14]. This work supports our previous work with MF and builds upon it to show Th17 induction in another role for these cells in gastric cancer. Given the abundance of MF in the GI tract, their role in inducing pro-inflammatory T cells should be closely examined in order to develop more effective treatments for inflammation associated cancers.
